# Omigapil Treatment Decreases Fibrosis and Improves Respiratory Rate in dy^2J^ Mouse Model of Congenital Muscular Dystrophy

**DOI:** 10.1371/journal.pone.0065468

**Published:** 2013-06-06

**Authors:** Qing Yu, Arpana Sali, Jack Van der Meulen, Brittany K. Creeden, Heather Gordish-Dressman, Anne Rutkowski, Sree Rayavarapu, Kitipong Uaesoontrachoon, Tony Huynh, Kanneboyina Nagaraju, Christopher F. Spurney

**Affiliations:** 1 Center for Genetic Medicine Research, Children’s National Medical Center, Washington DC, United States of America; 2 Division of Cardiology, Children’s National Medical Center, Washington DC, United States of America; 3 Kaiser SCPMG, Cure CMD, Olathe, Kansas, United States of America; University of Edinburgh, United Kingdom

## Abstract

**Introduction:**

Congenital muscular dystrophy is a distinct group of diseases presenting with weakness in infancy or childhood and no current therapy. One form, MDC1A, is the result of laminin alpha-2 deficiency and results in significant weakness, respiratory insufficiency and early death. Modification of apoptosis is one potential pathway for therapy in these patients.

**Methods:**

dy^2J^ mice were treated with vehicle, 0.1 mg/kg or 1 mg/kg of omigapil daily via oral gavage over 17.5 weeks. Untreated age matched BL6 mice were used as controls. Functional, behavioral and histological measurements were collected.

**Results:**

dy^2J^ mice treated with omigapil showed improved respiratory rates compared to vehicle treated dy^2J^ mice (396 to 402 vs. 371 breaths per minute, p<0.03) and similar to control mice. There were no statistical differences in normalized forelimb grip strength between dy^2J^ and controls at baseline or after 17.5 weeks and no significant differences seen among the dy^2J^ treatment groups. At 30–33 weeks of age, dy^2J^ mice treated with 0.1 mg/kg omigapil showed significantly more movement time and less rest time compared to vehicle treated. dy^2J^ mice showed normal cardiac systolic function throughout the trial. dy^2J^ mice had significantly lower hindlimb maximal (p<0.001) and specific force (p<0.002) compared to the control group at the end of the trial. There were no statistically significant differences in maximal or specific force among treatments. dy^2J^ mice treated with 0.1 mg/kg/day omigapil showed decreased percent fibrosis in both gastrocnemius (p<0.03) and diaphragm (p<0.001) compared to vehicle, and in diaphragm (p<0.013) when compared to 1 mg/kg/day omigapil treated mice. Omigapil treated dy^2J^ mice demonstrated decreased apoptosis.

**Conclusion:**

Omigapil therapy (0.1 mg/kg) improved respiratory rate and decreased skeletal and respiratory muscle fibrosis in dy^2J^ mice. These results support a putative role for the use of omigapil in laminin deficient congenital muscular dystrophy patients.

## Introduction

The congenital muscular dystrophies (CMDs) are a group of heterogeneous pediatric neuromuscular diseases that present with hypotonia, progressive scoliosis, contractures and respiratory insufficiency. [1] Currently recognized as a group of distinct diseases, the CMDs are a progressive and life limiting neuromuscular condition and there are currently no treatments available. The CMDs develop early and progressive respiratory insufficiency, with morbidity and mortality largely influenced by optimal pulmonary management. MDC1A, one type of CMD, is caused by defects in the laminin α2 gene (LAMA2) gene. [2] It is a severe and incapacitating disease with neonatal hypotonia, weakness, white matter changes and early death. [3] Histopathological analysis of these patients show significant muscle degeneration and increased apoptosis. [4] There are two current mouse models of laminin mutations used for preclinical studies, dy^W^ and dy^2J^. The dy^W^ mouse model demonstrates a severe phenotype with poor growth and early death due to absence of the laminin α2 protein. [5] The dy^2J^ model has a milder phenotype with a longer lifespan due to the presence of a truncated laminin α2 protein. [6] Both models display hindlimb paralysis related to demyelination and dystrophic changes in the skeletal muscle.

Recognition and lack of treatment for CMD patients has generated a need for further preclinical drug testing in CMD mouse models. Miyagoe et al. (1997) described a LAMA2 deficient mouse model (dy3K) with increased TUNEL positive nuclei in degenerating skeletal muscles in LAMA2 knockout mice. [7] Girgenrath et al. (2004) showed improved survival and myofiber histology after applying anti-apoptotic breeding crosses. [8] Dominov et al. (2005) also demonstrated increased growth and survival in LAMA2 null dy^W^ mice with over expression of the anti-apoptotic protein BCL2. [9] Most recently, Erb et al. (2009) demonstrated a role for the GADPH-Siah1-CBP/p300 apoptosis pathway in dy^W^ mice by demonstrating a beneficial effect on histology, locomotion, skeletal deformities, weight and survival in mice treated with omigapil. [10] This drug was also effective in a mouse model of progressive motor neuropathy. [11] Omigapil (TCH346) was used previously in clinical trials for Parkinson disease and amyotrophic lateral sclerosis where apoptosis is considered a key pathogenic pathway based on animal models. [12], [13] While neither trial demonstrated a clinical effect in these diseases, omigapil was well tolerated and may benefit patients with other neuromuscular diseases.

In this study, a phenotypic analysis of preclinical outcomes measures was performed in the dy^2J^ mouse model with truncated laminin α2 protein. These mice were treated with the anti-apoptotic agent omigapil at two doses to assess effects on outcome measures. dy^2J^ mice demonstrated functional and histological improvements and these results provide preclinical data for future putative clinical trials in CMD patients.

## Methods

### Animals

The protocol was approved and all mice were handled according to the local Institutional Animal Care and Use Committee guidelines (DCVAMC #01079). Generally, homozygous B6.WK-*Lama2^dy-2J^*/J (dy^2J^) and C57BL/6J (BL6) mice were purchased from the Jackson Laboratory (Bar Harbor, MA). All mice were housed in an individually vented cage system with a 12 hour light-dark cycle and received standard mouse chow and purified water *ad libitum*. All mice were acclimated, first to the room and then to different instruments for functional tests before the trial. Functional data was collected in the morning hours over a 2 week period. Mice were treated with omigapil (1 mg/kg/day or 0.1 mg/kg/day) daily via oral gavage starting at 12 to 15 weeks of age. Mice were treated for 10 weeks continuously and outcome data collected. Mice received no omigapil for the following 4 weeks and outcome data was collected. Mice were then retreated with omigapil for another 3.5 weeks and final measurements obtained after 17.5 weeks.

### Grip Strength Test

Grip strength was assessed using a grip strength meter consisting of horizontal forelimb mesh and an angled hind limb mesh (Columbus Instruments, Columbus OH) as described previously. [14] For forelimb strength, the animals were held so that only the forelimb paws grasped the flat mesh assembly and pulled back until their grip was broken. Five successful forelimb strength measurements within 2 minutes were recorded and the maximum values of each day over 5 day period were used for analysis.

### Open Field Activity (Digiscan)

Locomotor activity as measured using an open field digiscan apparatus (Omnitech Electronics, Columbus, OH) as previously described. [14] A total of 21 measurements per mouse including horizontal activity, vertical activity, total distance, movement time and rest time were recorded every 10 minutes for 1-hour as described previously.[15]–[17].

### Whole Body Plethysmography

The whole body plethysmography system (ADInstruments, St. Paul, MN) utilized a custom mouse chamber developed by the Research Instrument Shop at the University of Pennsylvania to minimize dead space. Other components include the spirometer (ML141), respiratory flow head (MLTL1) and the PowerLab 4/30 with LabChart software. The mouse was brought to the measurement room 15 minutes before the start of the measurement session to recover from the transportation and new environment stresses. The spirometer was calibrated every time the hardware was powered on to read in terms of flow (ml/s) rather than pressure (mv).Calibration of the plethysmography with 1ml of air was injected into the animal chamber to correlate the injected volume (ml) with the differential pressure (mv) measured in the chamber by integration. A 700 ml/min flow of dry air through the chambers was constantly delivered to avoid CO_2_ and water accumulation and to maintain a constant temperature. The mouse was weighed and placed into the mouse chamber first to acclimate for 10 minutes then record the respiratory flow data for 15 minutes. For data analysis, we calculated the values for respiratory rate using LabChart software.

### High Frequency Echocardiography

Echocardiography (VisualSonics Vevo 770, Toronto, Canada) was performed as detailed previously. [14] Mice were first anesthetized with 5% isoflurane mixed with 100% oxygen at 0.6 L/min flow, then maintained under anesthesia with 1.5% isoflurane/oxygen flow. A heating lamp was used to keep the heart rate and temperature constant at physiological status. Heart rate, aortic/pulmonary velocity, fractional shortening (FS), ejection fraction (EF) and mitral valve (MV) E/A ratio were obtained for cardiac function assessment. Qualitative and quantitative measurements were made offline using analytic software.

### In vitro Muscle Force Testing

*In vitro* muscle force testing (Servomotor/force transducer model 305B, Aurora Scientific, Ontario, Canada) was performed as previously described. [18] Briefly, the extensor digitorum longus (EDL) muscle from the right hindlimb was carefully dissected from the mouse and placed in an *in vitro* bath filled with Ringer solution. The maximal force generated by the muscle was measured at the determined optimal length.

### Histological Evaluations

Paraffin sections and H&E staining were performed by Histoserv, Inc. (Germantown, MD). Ten non-overlapping representative fields of the tissue were imaged under a light microscope at an objective of 40X and a digital image obtained using computer software (Olympus C.A.S.T. Stereology System, Olympus America Inc., Center Valley, PA). The digital images were loaded into Image J (NIH) for counting total fiber, regenerated fiber, fiber with centralized nuclei and percent centralized nuclei fiber was calculated. [14] The measurements were averaged from 10 non-overlapping fields. Due to extensive areas of degenerating fibers in dy^2J^ mice, images of the gastrocnemius were also obtained at 10X and used to determine percent area of degenerating fibers. Areas of degeneration were outlined using Image J program and compared to total tissue area for 10 non-overlapping areas. The calculated areas were averaged and expressed as percent area of degeneration. Apoptotic myonuclei were detected in frozen sectioned tibialis anterior muscle by TACS 2 TdT-DAB labeling (TUNEL) with use of the In Situ apoptosis detection kit (Trevigen, Gaithersburg, MD). Each group had 4 samples for the TUNEL assay. BL6 slides treated with TACS-Nuclease (Trevigen, Gaithersburg, MD) were used as positive controls. Six non-overlapping fields of the entire tissue section were imaged at 40X. Only TUNEL- and Methyl Green-positive nuclei that were located within muscle fibers were counted as apoptotic myonuclei. Apoptotic myonuclei values were averaged and expressed as percentage relative to the total number of myonuclei.

### Quantification of Fibrosis

Paraffin sections of gastrocnemius and diaphragm tissue were stained with picrosirius red by Histoserv, Inc. (Germantown, MD). The tissues were magnified under a light microscope at an objective of 1.25X and digital images obtained using computer software (Olympus C.A.S.T. Stereology System, Olympus America Inc., Center Valley, PA). These digital images were processed using Image J (NIH) with additional threshold color plug-ins to process jpeg images. Pixels corresponding to the area stained in red were normalized to the total pixel area of the tissue image and the results were expressed as percent of collagen. [18].

### Statistical Analysis

Normality of each quantitative measurement was assessed using the Shapiro-Wilk normality test and those measurements not meeting the normality assumption were analyzed with nonparametric tests. Mean comparisons between treatment groups were done at baseline (Table 1) and at 17.5 weeks (Table 2) using analysis of variance (ANOVA). For those ANOVA models showing a significant overall p-value (p<0.05), *post-hoc* pair-wise linear tests were performed with the resulting p-values adjusted for multiple testing using the Sidak method. Median comparisons between treatment groups were done for those non-normally distributed measures (open field activity) using Kruskal-Wallis tests. For those tests showing a significant p-value (p<0.05), *post-hoc* pair-wise linear tests were performed using Wilcoxon rank sum tests with the resulting p-values adjusted for multiple testing using the Sidak method. Histological evaluations were compared between groups using poisson regression for count data with group included as an indicator variable. Measurements at 17.5 weeks were also evaluated as a percentage of mean W/T values where percentage was calculated as (individual values/mean of W/T group) * 100. Median percentages were compared between three dy^2J^ mice groups using Kruskal-Wallis tests with *post-hoc* pairwise comparisons done with Wilcoxon rank sum tests and resulting p-values adjusted using the Sidak method. The percentage of W/T could not be calculated for several histological evaluations could due to all W/T animals having a zero value. Nominal significance was set at alpha = 0.05 and all analyses were performed using Stata V 11 (College Station, TX).

**Table 1:** Baseline outcome measures for BL6 control and dy^2J^ mice at 12–15 weeks of age show decreased body weights, forelimb grip strength, vertical activity and increased heart rates in dy^2J^ mice.

Measurement	BL6		dy^2J^		P-value
	N	Mean ± SD	N	Mean ± SD	
%FS	6	34±1	10	33±1	0.1421
%EF	6	63±1	10	63±1	0.9125
Heart rate (BPM)	6	459±23	10	524±18	<0.001
PA velocity (mm/s)	6	757±72	10	741±59	0.6365
Ao velocity (mm/s)	6	1058±101	10	1070±68	0.7868
E/A ratio	6	1.78±0.06	9	1.70±0.11	0.1060
Horizontal activity*	6	1510±564; 1578 (712–2390)	21	1286±247; 1213 (954–1784)	0.2938
Total distance (cm)*	6	431±219; 389 (156–811)	21	338±144; 304 (115–671)	0.2678
Movement time(second)*	6	52±27; 48 (19–99)	21	47±19; 45 (18–92)	0.7483
Rest time(second)*	6	548±27; 553 (501–581)	21	553±19; 555 (508–582)	0.7263
Vertical activity*	6	27±9; 25 (16–41)	21	6±4; 5 (0–15)	0.0002
GSM forelimb (KGF)	6	0.112±0.014	21	0.073±0.010	<0.001
Normalized GSM forelimb (KGF/kg)	6	4.519±0.871	21	3.973±0.664	0.1095
Body weight (g)	6	25.1±3.7	21	18.6±1.8	<0.001

* Non-parametric comparison of medians; data expressed as mean ± SD; median (range).

Abbreviations: %FS – percent fractional shortening, %EF- percent ejection fraction, BPM- beats per minute, Om – omigapil, SD – standard deviation, PA – pulmonary artery, Ao – aortic, E/A – ratio of mitral valve E and A wave velocities, GSM – grip strength meter, KGF – kilogram-force.

**Table 2:** Outcome measures for BL6 control and vehicle treated dy^2J^ mice at 30–33 weeks of age show decreased activity, in vitro force and fibrosis.

Measurement	BL6 Control		dy^2J^ vehicle		P-value
	N	Mean ± SD	N	Mean ± SD	
%FS	6	34±1	7	34±1	0.4952
%EF	6	63±2	7	64±2	0.2857
Heart rate (BPM)	6	475±9	7	548±18	<0.001
PA velocity (mm/s)	6	723±50	7	724±88	0.8109
Ao velocity (mm/s)	6	1039±79	7	924±105	0.0514
E/A ratio	6	1.65±0.07	7	1.66±0.05	0.7022
Horizontal activity*	6	1436±653; 1661 (598–2046)	7	619±111; 562 (487–748)	0.0321
Total distance (cm)*	6	328±208; 387 (62–539)	7	63±20; 70 (34–84)	0.0152
Movement time(second)*	6	38±23; 44 (8–59)	7	9±3; 10 (5–13)	0.0179
Rest time(second)*	6	562±23; 557 (541–592)	7	591±3; 590 (587–595)	0.0179
Vertical activity*	6	17±8; 20 (6–25)	7	0±0; 0 (0–0)	0.0011
GSM forelimb (KGF)	6	0.132±0.014	7	0.090±0.010	<0.001
Normalized GSM forelimb (KGF/kg)	6	4.440±1.205	7	4.239±0.483	0.6917
Body weight (g)	6	31.2±7.0	7	21.4±2.6	0.0052
Respiratory rate (bpm)	6	405±6	7	371±18	0.0010
Heart weight/BW	6	3.78±0.60	6	4.34±0.36	0.0757
Spleen weight/BW	6	3.20±1.18	7	3.34±0.55	0.7785
Gastroc Weight/BW	6	4.35±0.56	7	2.25±0.47	<0.001
Soleus weight/BW	6	0.33±0.04	7	0.24±0.10	0.0655
TA weight/BW	6	1.56±0.23	6	1.08±0.32	0.0128
Hindlimb maximal force	6	414±71	7	223±31	<0.001
Hindlimb specific force	6	255±32	7	184±28	0.0013
% fibrosis – gastroc	6	2.1±0.2	7	20.6±2.6	<0.001
% fibrosis - diaphragm	6	8.4±1.8	7	14.7±0.7	<0.001
% area with degenerating fibers – gastroc	6	0±0	7	10.1±3.10	<0.001
% Centralized nuclei fiber-gastroc*	6	2.8±1.0; 2.5 (1.9–4.6)	7	36.4±3.2; 38.0 (30.5–39.5)	0.0027
% Centralized nuclei fiber-diaphragm *	6	2.9±0.3; 3.0 (2.5–3.2)	7	13.6±1.5; 13.3 (12.0–16.6)	0.0027
% apoptosis nuclei per field*	4	0.7±0.7; 0.7 (0.4–1.0)	3	36.2±4.1; 38.0 (31.5–39.2)	0.0339

* Non-parametric comparison of medians; data expressed as mean ± SD; median (range).

Abbreviations: %FS – percent fractional shortening, %EF- percent ejection fraction, BPM- beats per minute, bpm – breaths per minute, SD – standard deviation, PA – pulmonary artery, AO – aortic, E/A – ratio of mitral valve E and A wave velocities, GSM – grip strength meter, BW- body weight, Gastroc – gastrocnemius, TA – tibialis anterior, KGF – kilogram-force.

## Results

### Phenotypic Differences Between dy^2J^ Mice and C57BL6/J Control Mice

#### Body and organ weights

At 12–15 weeks of age, dy^2J^ mice were significantly smaller than control mice (Table 1). From this 12–15 to 30–33 weeks of age, control mice increased their weight by 20% and dy^2J^ increased by only 10% (Table 2). dy^2J^ mice had consistently lower normalized tissue weights compared to controls for the gastrocnemius, tibialis anterior and soleus. The heart and spleen were slightly increased but not significantly different.

#### Behavioral assessment

dy^2J^ mice had significantly lower maximal forelimb grip strength when compared to the BL6 control mice at 12–15 and 30–33 weeks of age. However, when normalized for body weight, there were no statistical differences. Hindlimb grip strength was not performed since the hindlimbs showed significant atrophy due to paralysis throughout the duration of the study. There were no significant differences in horizontal activity, total distance, movement time or rest time between control and dy^2J^ mice at 12–15 weeks of age, but dy^2J^ mice showed significantly decreased vertical activity. At 30–33 weeks of age, dy^2J^ mice showed significantly decreased horizontal activity, total distance, movement time, rest time and vertical activity compared to controls.

#### Functional assessment

Lung function was assessed by whole body plethysmography at 30–33 weeks of age. dy^2J^ mice showed significantly decreased respiratory rates compared to control mice. There were no significant differences in cardiac function between dy^2J^ and control mice. Both strains showed normal systolic function at 12–15 and 30–33 weeks of age. However, dy^2J^ mice had significantly increased heart rates compared to control mice. dy^2J^ mice had significantly lower hindlimb EDL maximal and specific force when compared to the control group at 30–33 weeks of age.

Longitudinal changes in selected outcome measures are shown in Figure S1. Individual measures for selected outcomes and age of measurement are shown in Figure S2.

#### Histological assessment

**Figure 1:**
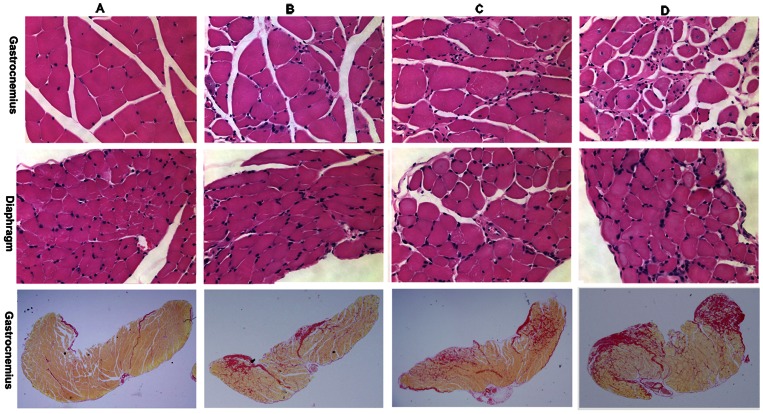
Histological analysis of gastrocnemius and diaphragm with H&E (top two rows) and gastrocnemius with picrosirius red (bottom row) show increased fibrosis and centralized nuclei in dy^2J^ mice. BL6 control mice are shown in column A. dy^2J^ mice treated with 0.1 mg/kg omigapil (Column B) showed markedly less fibrosis compared to dy^2J^ mice treated with 1 mg/kg omigapil (Column C) or vehicle (Column D).

dy^2J^ mice had significantly increased percent fibrosis in the gastrocnemius and diaphragm when compared to the BL6 control at 30–33 weeks of age. dy^2J^ mice also demonstrated significantly increased percent area of degenerating fibers in the gastrocnemius and increased percent of centralized nuclei per fiber in the gastrocnemius and diaphragm. (Figure 1).

#### TUNEL assay

**Figure 2:**
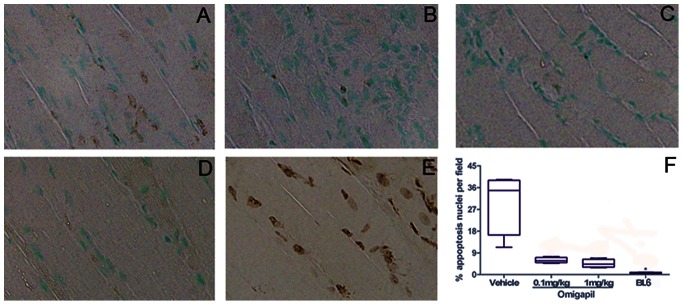
Apoptosis analysis of frozen tibialis anterior muscle with TUNEL assay on vehicle (A), Omigapil 0.1 mg/kg (B), Omigapil 1 mg/kg (C) treated dy^2J^ and BL6 control (D) groups show vehicle treated dy^2J^ mice had significantly more apoptosis than BL6 control (A, D, F; *P<0.05). Both Omigapil treated groups showed less apoptosis than vehicle treated group although statistically not different (A-C, F). BL6 slide treated with TACS-Nuclease was used as positive control (E).

In the tibialis anterior, there was a significant increase in the percent TUNEL positive nuclei per field found in dy^2J^ mice compared to controls (p<0.04). (Figure 2).

#### Analysis of values as a percentage of mean wild type values

Table S1 demonstrates how the outcome measures in dy^2J^ mice vary in respect to wild type mice measures at 30–33 weeks of age. The previous results are depicted as a percentage of the wild type value and show decreased body/organ weights, activity levels, grip strength and specific force measures.

### Phenotypic Differences Between dy^2J^ Mice Treated with Omigapil and Untreated dy^2J^ Mice

At 30–33 weeks of age, there were no significant differences in body weights, organ weights or grip strength among the three dy^2J^ homozygous groups with different omigapil dosages and vehicle treatment (Table 3). Outcome measures for controls and vehicle and treated dy^2J^ mice at 22–25 weeks of age and 26–29 weeks of age are shown in Table S2 and S3.

**Table 3:** Outcome measures for 30–33 week old omigapil and vehicle treated dy^2J^ mice after 17.5 weeks show significant improvements in respiratory rate and fibrosis in dy^2J^ 0.1 mg/kg omigapil treated mice.

Measurement	dy^2J^ vehicle		dy^2J^ Omigapil 0.1 mg		dy^2J^ Omigapil 1 mg		Significantly different mean/medians
	N	Mean ± SD	N	Mean ± SD	N	Mean ± SD	
%FS	7	34±1	7	33±3	7	34±1	NONE
%EF	7	64±2	7	63±2	7	64±2	NONE
Heart rate (BPM)	7	548±18	7	527±34	7	563±63	NONE
PA velocity (mm/s)	7	724±88	7	694±74	7	687±93	NONE
Ao velocity (mm/s)	7	924±105	7	917±106	7	942±73	NONE
E/A ratio	7	1.66±0.05	7	1.68±0.11	5	1.81±0.15	NONE
Horizontal activity*	7	619±111; 562 (487–748)	7	906±290; 778(682–1332)	7	696±258; 802 (203–1285)	NONE
Total distance (cm)*	7	63±20; 70 (34–84)	7	163±110; 112(69–324)	7	128±97; 115 (15–296)	NONE
Movement time(second)*	7	9±3; 10 (5–13)	7	23±14; 17 (11–45)	7	19±14; 18 (2–43)	Vehicle vs. 0.1 mg (p = 0.026)
Rest time(second)*	7	591±3; 590 (587–595)	7	577±14; 583(555–589)	7	581±14; 582 (557–598)	Vehicle vs. 0.1 mg (p = 0.026)
Vertical activity*	7	0±0; 0 (0–0)	7	0.1±0.4; 0 (0–1)	7	0±0; 0 (0–0)	NONE
GSM forelimb (KGF)	7	0.090±0.010	7	0.084±0.012	7	0.086±0.010	NONE
Normalized GSM forelimb(KGF/kg)	7	4.239±0.483	7	4.318±0.603	7	4.321±0.621	NONE
Body weight (g)	7	21.4±2.6	7	19.5±2.3	7	20.3±3.2	NONE
Respiratory rate (bpm)	7	371±18	7	396±11	7	402±19	Vehicle vs. 0.1 mg (p = 0.026)Vehicle vs. 1 mg (p = 0.006)
Heart weight/BW	6	4.34±0.36	7	4.38±0.40	7	4.51±0.53	NONE
Spleen weight/BW	7	3.34±0.55	7	3.13±0.63	7	3.22±0.62	NONE
Gastroc Weight/BW	7	2.25±0.47	7	2.19±0.58	7	2.17±0.48	NONE
Soleus weight/BW	7	0.24±0.10	7	0.28±0.05	7	0.30±0.07	NONE
TA weight/BW	6	1.08±0.32	7	1.28±0.18	7	1.28±0.27	NONE
Hindlimb maximal force	7	223±31	7	220±41	7	223±46	NONE
Hindlimb specific force	7	184±28	7	193±27	7	177±22	NONE
% fibrosis – gastroc	7	20.6±2.6	7	16.4±2.0	7	17.2±3.3	Vehicle vs. 0.1 mg (p = 0.030)
% fibrosis - diaphragm	7	14.7±0.7	7	9.4±1.7	7	12.2±2.1	Vehicle vs. 0.1 mg (p<0.001)Vehicle vs. 1 mg (p = 0.032)0.1 mg vs. 1 mg (p = 0.013)
% area with degeneratingfibers – gastroc.	7	10.1±3.1	7	3.0±0.7	7	5.8±3.8	Vehicle vs. 0.1 mg (p = 0.001)Vehicle vs. 1 mg (p = 0.037)
% Centralized nucleifiber-gastroc*	7	36.4±3.2; 38.0 (30.5–39.5)	7	28.2±3.0; 27.3 (25.3–33.4)	7	29.0±4.9; 27.6 (22.7–36.1)	Vehicle vs. 0.1 mg (p = 0.0120)Vehicle vs. 1 mg (p = 0.0376)
% Centralized nucleifiber-diaphragm*	7	13.6±1.5; 13.3 (12.0–16.6)	6	13.2±1.9; 12.9 (11.1–16.5)	7	12.8±1.5; 13.1 (10.9–14.9)	NONE
% apoptosis nuclei per field*	3	36.3±4.1; 38.0 (31.5–39.2)	4	5.9±1.1; 5.6 (4.7–7.5)	4	4.5±1.7; 4.3 (2.9–6.9)	NONE

* Non-parametric comparison of medians; data expressed as mean ± SD; median (range).

Abbreviations: %FS – percent fractional shortening, %EF- percent ejection fraction, BPM- beats per minute, bpm – breaths per minute, SD – standard deviation, PA – pulmonary artery, AO – aortic, E/A – ratio of mitral valve E and A wave velocities, GSM – grip strength meter, BW- body weight, Gastroc – gastrocnemius, TA – tibialis anterior, KGF.

#### Behavioral assessments

At the completion of the trial, dy^2J^ mice treated with 0.1 mg/kg omigapil showed significantly more movement time and less rest time when compared to vehicle treated dy^2J^ mice. There were no significant differences seen in other parameters, although the values for the dy^2J^ mice were decreased for all parameters and only showed slight improvements with omigapil treatment.

#### Functional assessments

At the completion of the trial, dy^2J^ mice treated with 0.1 mg/kg and 1 mg/kg omigapil showed significantly increased respiratory rates compared to vehicle treated dy^2J^ mice. Respiratory rates for omigapil treated mice were similar to control mice. Treatment with omigapil did not alter cardiac function or in vitro force testing.

Longitudinal changes in selected outcome measures are shown in Figure S1. Individual measures for selected outcomes and age of measurement are shown in Figure S2.

#### Histological assessment

In the gastrocnemius, the dy^2J^ group treated with 0.1 mg/kg omigapil showed significantly decreased fibrosis compared to the vehicle treated dy^2J^ mice. dy^2J^ mice treated with 1 and 0.1 mg/kg omigapil showed significantly decreased fibrosis in the diaphragm compared to the vehicle and the 0.1 mg/kg omigapil treated dy^2J^ mice were also significantly decreased compared to the 1 mg/kg treatment group. Both 1 and 0.1 mg/kg omigapil treatment led to a significant decrease in the percent area of degenerating fibers and percent centralized nuclei per fiber in the gastrocnemius compared to vehicle treated mice. (Figure 1).

#### TUNEL assay

There was a decrease in the percent TUNEL positive nuclei per field in omigapil treated dy^2J^ mice. The differences between vehicle and each of the treatments are significant alone, but when adjusted for multiple comparisons by comparing each group to the others, they do not reach significance. (Figure 2).

#### Analysis of values as a percentage of mean wild type values

Table S1 demonstrates how the outcome measures in dy^2J^ mice vary in respect to wild type mice measures at the end of the trial. Consistent with the data analysis, the dy^2J^ mice treated with 0.1 mg/kg/day omigapil showed significantly less decreased respiratory rate and less increased fibrosis in both gastrocnemius and diaphragm when compared to vehicle treated mice. The dy^2J^ mice treated with 1 mg/kg/day omigapil also showed significantly less decreased respiratory rate when compared to vehicle treated mice.

## Discussion

This study provides phenotypic data on the dy^2J^ mouse model of congenital muscular dystrophy and demonstrates effective outcome measures for preclinical trials. dy^2J^ mice demonstrated decreased body weights and poor growth, decreased forelimb grip strength, decreased respiratory rates and increased fibrosis in the gastrocnemius and diaphragm compared to control mice.

Apoptosis is one mechanism shown to be involved in the pathogenesis of MDC1A. [7]–[10] Omigapil, an inhibitor of the GAPDH-Siah1-mediated apoptosis, was found to be effective in the dy^W^ mouse model. [10] This study also demonstrated significant improvement in functional and histological measures in the dy^2J^ model after therapy with the omigapil (0.1 mg/kg), providing further support for clinical trials of omigapil in congenital muscular dystrophy.

Due to the mild phenotype of the dy^2J^ model, histological evaluation of fibrosis provided the strongest evidence for a beneficial effect of omigapil. Omigapil significantly reduced the percent of fibrosis in the gastrocnemius and the diaphragm. Erb et al. (2009) studied omigapil in the dy^W^/dy^W^ mouse model of congenital muscular dystrophy. [10] dy^W^ mice have a more severe phenotype than the dy^2J^ mice and early death. Erb et al. (2009) demonstrated decreased fibrosis of the triceps brachii with 0.1 mg/kg omigapil dosing. In our study, both the gastrocnemius and diaphragm demonstrated significantly decreased fibrosis in the 0.1 mg/kg omigapil group and just the diaphragm in the 1 mg/kg group. Decreased fibrosis is likely indicative of decreased muscle cell apoptosis due to omigapil therapy. In support of this, we also found decreased apoptosis in the tibialis anterior muscle of omigapil treated dy^2J^ mice. (Figure 2) Apoptosis is a known pathologic pathway in congenital muscular dystrophy patients. [4].

Erb et al. (2009) also measured manual recordings of mouse activity in a new cage environment and showed omigapil treated mice had significantly increased activity compared to vehicle treated mice at 5–6 weeks of age. This significance was lost at 10 weeks of age, but a trend continued. In the milder phenotype of the dy^2J^ mice, this study showed significantly increased movement times and decreased rest times in mice treated with 0.1 mg/kg. So, in the more severe model, an improvement was demonstrated early and lost over time, while in this milder phenotypic model, the improvements were beginning to show and likely require a longer treatment period to fully develop.

Erb et al. (2009) also presented histological data showing the muscle fiber size distribution normalized by reducing the proportion of small caliber and increasing the proportion of large caliber muscle fibers in the triceps brachii of dy^W^ mice treated with 0.1 mg/kg omigapil. The current study did not measure fiber size, but we did see a significant decrease in percent centralized nuclei per fiber (a measure of total regeneration) between omigapil treatment and vehicle control groups in the gastrocnemius. We also showed significantly decreased percent in areas of degenerating fibers in the gastrocnemius in the omigapil treated mice. A decrease in degeneration leads to less regeneration and preservation of larger fibers, a similar observation as reported by Erb et al.

dy^2J^ mice showed significantly increased respiratory rates in omigapil treated mice at the end of the trial compared to vehicle treated. These increased rates were similar to wild type controls. This *in vivo* functional measure could reflect improved diaphragm function. This finding is quite important since clinically many of the affected patients suffer significant respiratory insufficiency and this is a leading cause of death. Any effective therapy needs to demonstrate improvements in respiratory function and these changes support a putative role for omigapil.

Echocardiographic analysis found increased heart rates in dy^2J^ mice. This is a consistent finding in other dystrophic mouse models and could reflect imbalances in the sympathetic nervous system. [14] The increased heart rate does not appear to be a compensatory mechanism for decreased heart function, since the measurements of systolic function are normal. There was a trend towards normal heart rates in dy^2J^ mice treated with 0.1 mg/kg omigapil, but this did not reach significance.

Of interest, some of the functional data and the decreased fibrosis support a benefit of the lower dosing omigapil at 0.1 mg/kg compared to the higher dose at 1 mg/kg. While some of the body weight and activity data from Erb et al. (2009) support increased benefits from a higher dose of 1 mg/kg, Waldmeier et al. (2000) published dose response curves in other mouse models that showed benefits were lost above 1 mg/kg/day of omigapil. [19] Our study supports these findings, even showing that diaphragm fibrosis was significantly decreased in 0.1 mg/kg compared to 1 mg/kg omigapil dosing. This important preclinical observation might aid in the selection of drug dosing for clinical trials.

There were some limitations in this study. This study did not look at forelimb muscle force or histology. Due to the significant demyelination of the hindlimb and paralysis, hindlimb muscle changes were likely affected by mechanisms other than just laminin defects and may not reflect improvements seen in other muscle groups. This study also included a 10 week treatment period, followed by a 4 week washout period and then 3.5 weeks of retreatment. The purpose of this design was to uncover any significant drug effect with a washout period and if this effect was recoverable with retreatment. There were no significant differences shown in outcome measures after the initial 10 weeks of treatment (Table S2) or after the 4 week washout period (Table S3). This is potentially related to factors including minimal disease progression, variability in outcome measures and incomplete penetrance. As shown, significant findings were seen after the completion of 3.5 weeks of retreatment (Table 3). As noted, the major findings of the study were histological with trends towards functional improvements, but a longer treatment period will be required in future studies to reach significance.

In conclusion, this study shows decreased fibrosis and improved respiratory rate in dy^2J^ mice treated with 0.1 mg/kg omigapil. We provide phenotypic data to further characterize the dy^2J^ model for future preclinical trials. These findings support a putative role for omigapil in the treatment of congenital muscular dystrophy.

## Supporting Information

Figure S1 **Longitudinal functional test data for groups with mean and standard deviation for BL6 and dy^2J^ (vehicle, 0.1 mg/kg omigapil and 1 mg/kg omigapil) mice across the protocol time points: baseline; after 10 weeks of treatment with omigapil; after 4 week washout period; and after 3.5 weeks of retreatment with omigapil.**
(TIF)

Figure S2 **Individual mouse functional test data and age measured for BL6 and dy^2J^ (vehicle, 0.1 mg/kg omigapil, 1 mg/kg omigapil) mice during the main protocol time periods: baseline; after 10 weeks of treatment with omigapil; after 4 week washout period; and after 3.5 weeks of retreatment with omigapil.**
(TIF)

Table S1 **Analysis of outcome measure values as a percentage of mean wild type values in 30–33 week old omigapil and vehicle treated dy2J mice showing significance in respiratory rate and fibrosis.**
(DOCX)

Table S2 **Outcome measures for BL6 control, vehicle and Omigapil treated dy2J mice at 22–25 weeks of age when mice were treated for 10 weeks.**
(DOCX)

Table S3 **Outcome measures for BL6 control, vehicle and Omigapil treated dy2J mice at 26–29 weeks of age when mice stopped treatment for 4 weeks.**
(DOCX)
