# Supplementary figures and images for: Omigapil Treatment Decreases Fibrosis and Improves Respiratory Rate in dy2J Mouse Model of Congenital Muscular Dystrophy
Source: PLoS One. 2013 Jun 6;8(6):e65468. doi: 10.1371/journal.pone.0065468 (PMC3675144; doi:10.1371/journal.pone.0065468)

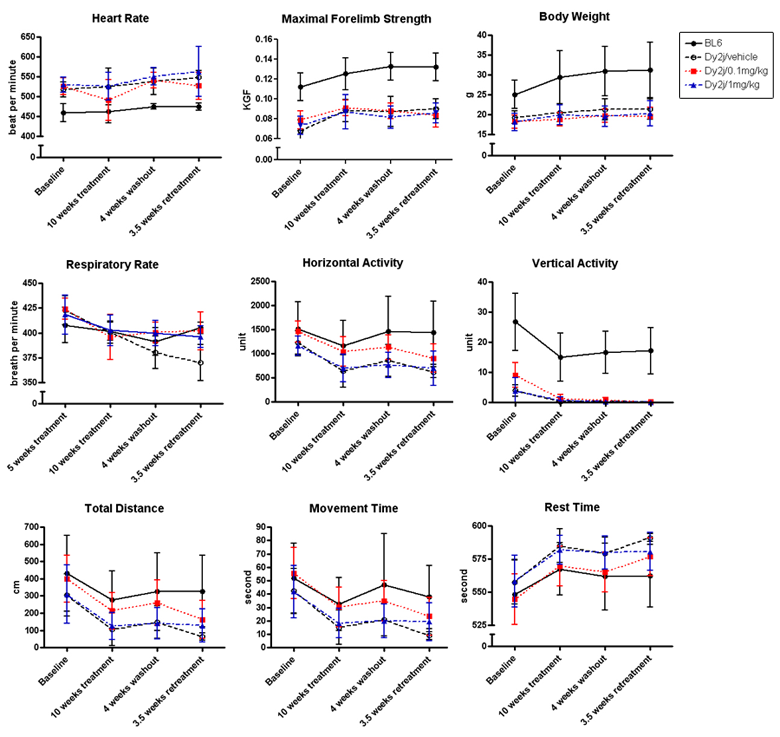

Supplement: Figure S1 — Longitudinal functional test data for groups with mean and standard deviation for BL6 and dy2J (vehicle, 0.1 mg/kg omigapil and 1 mg/kg omigapil) mice across the protocol time points: baseline; after 10 weeks of treatment with omigapil; after 4 week washout period; and after 3.5 weeks of retreatment with omigapil. (TIF) [file pone.0065468.s001.tif]

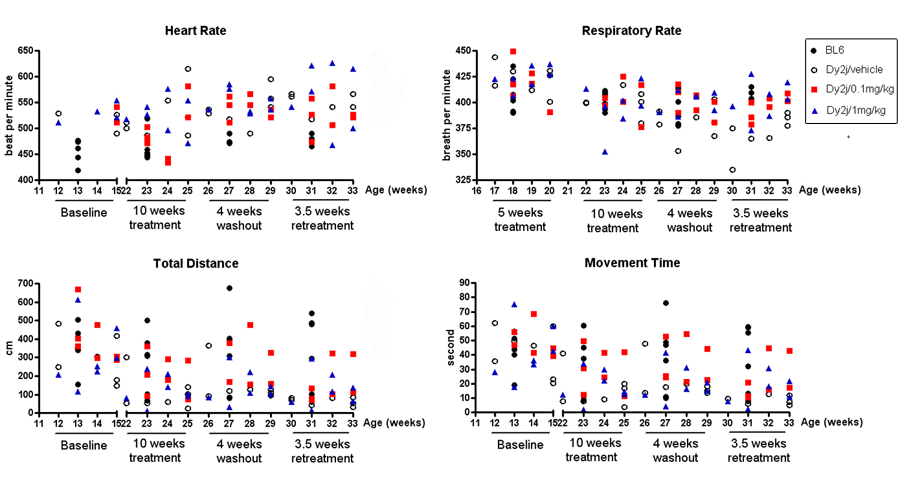

Supplement: Figure S2 — Individual mouse functional test data and age measured for BL6 and dy2J (vehicle, 0.1 mg/kg omigapil, 1 mg/kg omigapil) mice during the main protocol time periods: baseline; after 10 weeks of treatment with omigapil; after 4 week washout period; and after 3.5 weeks of retreatment with omigapil. (TIF) [file pone.0065468.s002.tif]
