# Supplementary material for: Omigapil Treatment Decreases Fibrosis and Improves Respiratory Rate in dy2J Mouse Model of Congenital Muscular Dystrophy
Source: PLoS One. 2013 Jun 6;8(6):e65468. doi: 10.1371/journal.pone.0065468 (PMC3675144; doi:10.1371/journal.pone.0065468)
Supplement: Table S1 — Analysis of outcome measure values as a percentage of mean wild type values in 30–33 week old omigapil and vehicle treated dy2J mice showing significance in respiratory rate and fibrosis. (DOCX) [file pone.0065468.s003.docx]

Table S1: Analysis of outcome measure values as a percentage of mean wild type values in 30-33 week old omigapil and vehicle treated dy^2J^ mice showing significance in respiratory rate and fibrosis

| **Parameter (as a % of W/T)*** | **Omigapil 0.1 mg** | | **Omigapil 1.0 mg** | | **Vehicle** | | **P-value (significantly different medians)** |
| --- | --- | --- | --- | --- | --- | --- | --- |
|  | **N** | **Mean ± SD;**  **Median (range)** | **N** | **Mean ± SD;**  **Median (range)** | **N** | **Mean ± SD;**  **Median (range)** |  |
| FS | 7 | 99 ± 3; 98 (95 – 104) | 7 | 101 ± 4; 101 (95 – 107) | 7 | 101 ± 3; 101 (98 – 107) | 0.252 |
| EF | 7 | 100 ± 2; 100 (97 – 103) | 7 | 102 ± 3; 100 (97 – 106) | 7 | 102 ± 3; 102 (98 – 106) | 0.405 |
| Heart rate | 7 | 111 ± 7; 111 (100 – 122) | 7 | 118 ± 13; 120 (98 – 132) | 7 | 116 ± 13; 114 (109 – 119) | 0.331 |
| PA velocity | 7 | 94 ± 10; 98 (75 – 105) | 7 | 94 ± 13; 92 (76 – 115) | 7 | 99 ± 12; 97 (81 – 114) | 0.780 |
| Ao velocity | 7 | 88 ± 10; 85 (77 – 109) | 7 | 91 ± 7; 91 (76 – 99) | 7 | 89 ± 10; 87 (81 – 109) | 0.483 |
| E/A ratio | 7 | 102 ± 7; 102 (94 – 114) | 7 | 109 ± 9; 107 (100 – 124) | 7 | 101 ± 3; 101 (96 – 104) | 0.118 |
| Horizontal activity | 7 | 63 ± 20; 54 (48 – 93) | 7 | 48 ± 25; 56 (14 – 90) | 7 | 43 ± 8; 39 (34 – 52) | 0.140 |
| Total distance | 7 | 50 ± 34; 34 (21 – 99) | 7 | 39 ± 30; 35 (5 – 90) | 7 | 19 ± 6; 21 (10 – 26) | 0.094 |
| Movement time | 7 | 62 ± 38; 45 (29 – 119) | 7 | 51 ± 38; 48 (5 – 114) | 7 | 24 ± 8; 27 (13 – 35) | 0.044 (NONE) |
| Rest time | 7 | 103 ± 3; 104 (99 – 105) | 7 | 103 ± 3; 103 (99 – 106) | 7 | 105 ± 3; 105 (104 – 105) | 0.044 (NONE) |
| GSM | 7 | 63 ± 9; 61 (52 – 77) | 7 | 65 ± 7; 65 (57 – 79) | 7 | 68 ± 8; 65 (57 – 79) | 0.620* |
| Normalized GSM | 7 | 97 ± 14; 96 (83 – 119) | 7 | 97 ± 14; 95 (82 – 122) | 7 | 95 ± 11; 92 (88 – 118) | 0.93 |
| BW | 7 | 62 ± 7; 60 (57 – 78) | 7 | 65 ± 10; 62 (52 – 79) | 7 | 68 ± 8; 68 (61 – 81) | 0.146 |
| Respiratory rate | 7 | 98 ± 3; 99 (94 – 101) | 7 | 99 ± 5; 99 (92 – 106) | 7 | 91 ± 5; 93 (83 – 96) | 0.009 (0.1mg vs. vehicle; p=0.021) (1mg vs. vehicle; p=0.038) |
| Heart weight/BW | 7 | 116 ± 11; 113 (103 – 136) | 7 | 120 ± 14; 117 (101 – 147) | 7 | 115 ± 9; 110 (108 – 131) | 0.542 |
| Spleen weight/BW | 7 | 98 ± 20; 105 (61 – 115) | 7 | 101 ± 19; 102 (68 – 127) | 7 | 104 ± 17; 103 (82 – 123) | 0.789 |
| Gastroc weight/BW | 7 | 50 ± 13; 48 (37 – 77) | 7 | 50 ± 11; 47 (34 – 64) | 7 | 52 ± 11; 54 (37 – 64) | 0.925 |
| Soleus weight/BW | 7 | 86 ± 16; 92 (61 – 104) | 7 | 92 ± 23; 101 (58 – 119) | 7 | 72 ± 31; 64 (37 – 129) | 0.293 |
| TA weight/BW | 7 | 82 ± 12; 83 (63 – 98) | 7 | 82 ± 17; 86 (45 – 96) | 7 | 69 ± 20; 71 (40 – 95) | 0.311 |
| Hindlimb Maximal force | 7 | 53 ± 10; 52 (40 – 73) | 7 | 54 ± 11; 53 (41 – 76) | 7 | 54 ± 7; 58 (38 – 59) | 0.619 |
| Hindlimb Specific force | 7 | 76 ± 11; 80 (58 – 85) | 7 | 69 ± 9; 70 (57 – 82) | 7 | 72 ± 11; 75 (55 – 85) | 0.409 |
| Fibrosis – gastroc | 6 | 759 ± 94; 785 (614 – 854) | 7 | 792 ± 153; 812 (605 – 1029) | 7 | 951 ± 121; 951 (803 – 1126) | 0.033 (0.1mg vs. vehicle; p=0.026) |
| Fibrosis – diaphragm | 6 | 112 ± 20; 106 (91 – 140) | 7 | 145 ± 25; 139 (107 – 179) | 7 | 174 ± 8; 175 (162 – 186) | 0.002 (0.1mg vs. vehicle; p=0.005) |
| % apoptosis nuclei per field | 3 | 5304 ± 606; 564 (4612 – 5728) | 4 | 867 ± 168; 841 (690 – 1094) | 4 | 670 ± 250; 625 (424 – 1006) | NONE |

Abbreviations: W/T – wild type, FS – percent fractional shortening, EF- percent ejection fraction, SD – standard deviation, PA – pulmonary artery, Ao – aortic, E/A – ratio of mitral valve E and A wave velocities, GSM – grip strength meter, BW- body weight, Gastroc – gastrocnemius, TA – tibialis anterior
