# Supplementary material for: Omigapil Treatment Decreases Fibrosis and Improves Respiratory Rate in dy2J Mouse Model of Congenital Muscular Dystrophy
Source: PLoS One. 2013 Jun 6;8(6):e65468. doi: 10.1371/journal.pone.0065468 (PMC3675144; doi:10.1371/journal.pone.0065468)
Supplement: Table S2 — Outcome measures for BL6 control, vehicle and Omigapil treated dy2J mice at 22–25 weeks of age when mice were treated for 10 weeks. (DOCX) [file pone.0065468.s004.docx]

Table S2: Outcome measures for BL6 control, vehicle and Omigapil treated dy^2J^ mice at 22-25 weeks of age when mice were treated for 10 weeks

| **Measurement** | **BL6** | | **dy2J vehicle** | | **dy2J Omigapil 0.1 mg** | | **dy2J Omigapil 1 mg** | | **P value BL6 Vs. dy2J vehicle** | **Significantly different among dy2J vehicle and Omigapil treated** |
| --- | --- | --- | --- | --- | --- | --- | --- | --- | --- | --- |
|  | **N** | **Mean ± SD** | **N** | **Mean ± SD** | **N** | **Mean ± SD** | **N** | **Mean ± SD** |  |  |
| %FS | 6 | 35±1 | 7 | 35 ± 1 | 7 | 35 ± 1 | 7 | 35 ± 1 | 0.668 | NONE |
| %EF | 6 | 65±2 | 7 | 66 ± 1 | 7 | 66 ± 2 | 7 | 65 ± 2 | 0.223 | NONE |
| Heart rate (BPM) | 6 | 462±28 | 7 | 525 ± 46 | 7 | 491 ± 51 | 7 | 526 ± 35 | 0.015 | NONE |
| PA velocity (mm/s) | 6 | 759 ± 114 | 7 | 680 ± 59 | 7 | 680 ± 54 | 7 | 667 ± 73 | 0.140 | NONE |
| Ao velocity (mm/s) | 6 | 1033±64 | 7 | 919 ± 113 | 7 | 880 ± 50 | 7 | 905 ± 116 | 0.051 | NONE |
| E/A ratio | 6 | 1.76±0.08 | 7 | 1.68 ± 0.07 | 7 | 1.72 ± 0.12 | 7 | 1.69 ± 0.14 | 0.087 | NONE |
| Horizontal activity* | 6 | 1159 ± 528 1317 (414-1692) | 7 | 642 ± 340 431 (315-1231) | 7 | 1043 ± 304 1143 (634-1427) | 7 | 704 ± 284 752 (166-1065) | 0.063 | NONE |
| Total distance (cm)* | 6 | 279 ± 166 312 (66 – 501) | 7 | 105 ± 94 59 (25-300) | 7 | 213 ± 107 208 (75-362) | 7 | 126 ± 78 105 (13-240) | 0.032 | NONE |
| Movement time(second)* | 6 | 33 ± 20 35 (9 – 60) | 7 | 15 ± 13 9 (4- 41) | 7 | 30 ± 15 31 (11 – 49) | 7 | 18 ± 11 15 (2 – 34) | 0.073 | NONE |
| Rest time(second)* | 6 | 568 ± 20 566 (540 – 591) | 7 | 584 ± 13 591 (599-596) | 7 | 570 ± 15 569 (551-589) | 7 | 582 ± 11 586(566-598) | 0.073 | NONE |
| Vertical activity* | 6 | 15 ± 8 17 (5 – 25) | 7 | 0.3 ± 0.5 0 (0 – 1) | 7 | 1.1 ± 1.6 0 (0 – 4) | 7 | 0.6 ± 1.1 0 (0 – 3) | 0.002 | NONE |
| GSM forelimb (KGF) | 6 | 0.125±0.016 | 7 | 0.088 ± 0.011 | 7 | 0.091 ± 0.008 | 7 | 0.087 ± 0.017 | <0.001 | NONE |
| Normalized GSM forelimb (KGF/kg) | 6 | 4.371±0.661 | 7 | 4.328 ± 0.504 | 7 | 4.878 ± 0.635 | 7 | 4.381 ± 0.596 | 0.892 | NONE |
| Body weight (g) | 6 | 29.4±6.7 | 7 | 20.5 ± 2.3 | 7 | 18.8 ± 1.7 | 7 | 19.9 ± 2.5 | 0.007 | NONE |
| Respiratory rate (bpm) | 6 | 402± 10 | 7 | 401± 11 | 7 | 403 ± 16 | 7 | 396 ± 23 | 0.897 | NONE |

* Non-parametric comparison of medians; data expressed as median (range)

Abbreviations: %FS – percent fractional shortening, %EF- percent ejection fraction, BPM- beats per minute, Om – omigapil, SD – standard deviation, PA – pulmonary artery, Ao – aortic, E/A – ratio of mitral valve E and A wave velocities, GSM – grip strength meter, KGF – kilogram-force
