# Supplementary material for: Omigapil Treatment Decreases Fibrosis and Improves Respiratory Rate in dy2J Mouse Model of Congenital Muscular Dystrophy
Source: PLoS One. 2013 Jun 6;8(6):e65468. doi: 10.1371/journal.pone.0065468 (PMC3675144; doi:10.1371/journal.pone.0065468)
Supplement: Table S3 — Outcome measures for BL6 control, vehicle and Omigapil treated dy2J mice at 26–29 weeks of age when mice stopped treatment for 4 weeks. (DOCX) [file pone.0065468.s005.docx]

Table S3: Outcome measures for BL6 control, vehicle and Omigapil treated dy^2J^ mice at 26-29 weeks of age when mice stopped treatment for 4 weeks

| **Measurement** | **BL6** | | **dy2J vehicle** | | **dy2J Omigapil 0.1 mg** | | **dy2J Omigapil 1 mg** | | **P value BL6 Vs. dy2J vehicle** | **Significantly different among dy2J vehicle and Omigapil treated** |
| --- | --- | --- | --- | --- | --- | --- | --- | --- | --- | --- |
|  | **N** | **Mean ± SD** | **N** | **Mean ± SD** | **N** | **Mean ± SD** | **N** | **Mean ± SD** |  |  |
| %FS | 6 | 34±1 | 7 | 36 ± 3 | 7 | 34 ± 1 | 7 | 34 ± 1 | 0.224 | NONE |
| %EF | 6 | 63±2 | 7 | 66 ± 4 | 7 | 64 ± 2 | 7 | 64 ± 2 | 0.094 | NONE |
| Heart rate (BPM) | 6 | 475±7 | 7 | 538 ± 33 | 7 | 541 ± 20 | 7 | 551 ± 22 | <0.001 | NONE |
| PA velocity (mm/s) | 6 | 734 ± 50 | 7 | 735 ± 48 | 7 | 716 ± 74 | 7 | 693 ± 76 | 0.962 | NONE |
| Ao velocity (mm/s) | 6 | 1049±41 | 7 | 844 ± 63 | 7 | 886 ± 72 | 7 | 881 ± 86 | <0.001 | NONE |
| E/A ratio | 6 | 1.72±0.11 | 7 | 1.72 ± 0.12 | 7 | 1.69 ± 0.13 | 5 | 1.73 ± 0.15 | 0.999 | NONE |
| Horizontal activity* | 6 | 1463 ± 731; 1666(535-2235) | 7 | 859 ± 329; 750(501-1553) | 7 | 1134 ± 253; 1026(863-1436) | 7 | 766 ± 262; 738(341-1156) | 0.199 | NONE |
| Total distance (cm)* | 6 | 325 ± 225; 352(81-677) | 7 | 148 ± 96; 119(93-363) | 7 | 261 ± 131; 168(154-475) | 7 | 141 ± 92; 108(30-301) | 0.391 | 0.1mg vs. Vehicle (p=0.038) |
| Movement time(second)* | 6 | 38 ± 25; 42(10-76) | 7 | 21 ± 12; 18(13-48) | 7 | 35 ± 15; 25(21-54) | 7 | 20 ± 13; 16(4-42) | 0.474 | 0.1mg vs. Vehicle (p=0.038) |
| Rest time(second)* | 6 | 562 ± 25; 559(524-590) | 7 | 579 ± 12; 582(552-587) | 7 | 565 ± 15; 575(546-579) | 7 | 580 ± 13; 584(558-596) | 0.474 | 0.1mg vs. Vehicle (p=0.038) |
| Vertical activity* | 6 | 17 ± 7; 18(7-27) | 7 | 0.4 ± 1.1; 0 (0 – 3) | 7 | 0.6 ± 0.8; 0 (0 – 2) | 7 | 0.1 ± 0.4; 0 (0 – 1) | 0.002 | NONE |
| GSM forelimb (KGF) | 6 | 0.133±0.014 | 7 | 0.087 ± 0.015 | 7 | 0.088 ± 0.009 | 7 | 0.082 ± 0.011 | <0.001 | NONE |
| Normalized GSM forelimb (KGF/kg) | 6 | 4.438±0.999 | 7 | 4.122 ± 0.798 | 7 | 4.489 ± 0.482 | 7 | 4.182 ± 0.375 | 0.540 | NONE |
| Body weight (g) | 6 | 30.9±6.2 | 7 | 21.3 ± 2.7 | 7 | 19.7 ± 1.7 | 7 | 19.6 ± 2.6 | 0.004 | NONE |
| Respiratory rate (bpm) | 6 | 391± 14 | 7 | 380± 16 | 7 | 400 ± 13 | 7 | 401 ± 10 | 0.218 | 0.1mg vs. Vehicle (p=0.038); 1.0mg vs. Vehicle (p=0.030) |

* Non-parametric comparison of medians; data expressed as mean ± SD; median (range)

Abbreviations: %FS – percent fractional shortening, %EF- percent ejection fraction, BPM- beats per minute, SD – standard deviation, PA – pulmonary artery, Ao – aortic, E/A – ratio of mitral valve E and A wave velocities, GSM – grip strength meter, KGF – kilogram-force
